# New karyologycal data and cytotaxonomic considerations on small mammals from Santa Virgínia (Parque Estadual da Serra do Mar, Atlantic Forest, Brazil)

**DOI:** 10.3897/CompCytogen.v8i1.6430

**Published:** 2014-01-24

**Authors:** Camilla Bruno Di-Nizo, Carolina Lima Neves, Júlio Fernando Vilela, Maria José de J. Silva

**Affiliations:** 1 Laboratório de Ecologia e Evolução, Instituto Butantan, Avenida Vital Brazil, 1500, CEP 05503-900, São Paulo, SP, Brazil; 2 Laboratório de Biologia da Conservação, Departamento de Ecologia, Universidade Estadual Paulista (UNESP), Avenida 24 A,1515. CEP 13506-900, Rio Claro, SP, Brazil; 3 Laboratório de Biologia Evolutiva Teórica e Aplicada, Universidade Federal do Rio do Janeiro (UFRJ), Avenida Professor Rodolpho Paulo Rocco, s/n – CCS, bl. A, sala A2-92. CEP 21941-617, Rio de Janeiro, RJ, Brazil

**Keywords:** Atlantic Forest, conservation, cytotaxonomy, *Monodelphis scalops*

## Abstract

Atlantic Forest, in the eastern coast of Brazil, is a hotspot of biodiversity of mammals, and Parque Estadual da Serra do Mar (PESM) is the largest continuous area of this biome. Here, we characterized the karyotype composition of the small mammals from Santa Virgínia, a region in the northern part of PESM. Specimens were collected from July 2008 to September 2009. We identified 17 species (13 rodents and 4 marsupials) from which 7 exhibited species-specific karyotypes, illustrating the importance of karyotype information in cytotaxonomy. We report for first time the karyotype of *Monodelphis scalops* (Thomas, 1888) and two new records for PESM: *Akodon montensis* Thomas, 1913 and *Brucepattersonius soricinus* Hershkovitz, 1998. Cytogenetic polymorphisms were detected for some species trapped in the area. Our results show the importance of Santa Virgínia / PESM in addressing studies for the conservation of small mammal wildlife in the Atlantic Forest.

## Introduction

The Atlantic Forest is the fourth biodiversity hotspot in the world (Myers et al. 2000, Ceballos and Ehrlich 2006, Carnaval et al. 2009). Geographical aspects combined with the large altitudinal and longitudinal ranges have favored the emergence of high endemism and species richness in this biome (Leal and De Gusmão Câmara 2003, Ribeiro et al. 2009). Nevertheless, the remaining forest represents only approximately 11% of the original extent, which highlights the biome as a priority for biodiversity conservation (Ribeiro et al. 2009).

The Parque Estadual da Serra do Mar (PESM), located in the state of São Paulo, Brazil was created in 1977, and is considered the largest remaining block of Atlantic Forest with 315.390 hectares (Instituto Florestal 2006).

Studies the mammal fauna of this park are scarce and the majority of the reports were presented in undergraduate theses and master’s dissertations, focusing on large mammals (Wang 2002, Norris et al. 2012). The most comprehensive article about small mammals from PESM was performed in Picinguaba (Northern of PESM) and reported morphology and karyotype information of 27 species belonging to the orders Didelphimorphia, Carnivora, and Rodentia (Pinheiro and Geise 2008).

According to Paglia et al. (2012), small mammals of the orders Rodentia and Didelphimorphia are important components of the Atlantic Forest mammal fauna, representing approximately 40% of the species. Morphological studies combined with cytogenetics and geographical distribution information allow the proper identification of taxa, particularly in cases of cryptic or morphologically similar species. Moreover, cytogenetic study can reveal genetic variability within and among individuals.

This study aims to characterize the karyotype composition and contribute to the identification of small rodents and marsupials from Santa Virgínia, since there is only one published study focusing on small mammals of this area. Data about geographical distribution of trapped species are also given.

## Material and methods

### Study area

**Figure 1.**
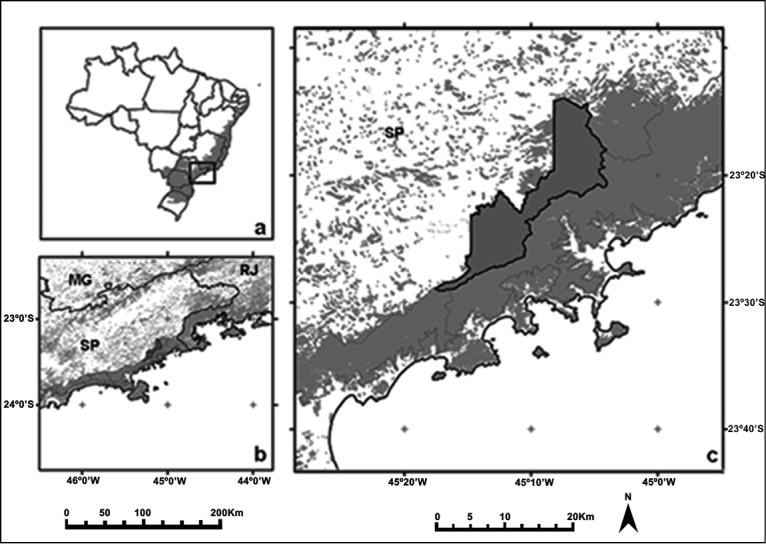
**a** Map of Brazil with original Atlantic Forest cover in grey and the region of Parque Estadual da Serra do Mar (PESM) indicated (square) **b** Parque Estadual da Serra do Mar (PESM) in grey **c** Santa Virgínia is highlighted (extracted and modified from Instituto Florestal 2006).

Santa Virgínia (lat. 23°24.00'S to 23°17.00'S, long. 45°03.00'W to 45°11.00'W) is located in the Northern of PESM (Fig. 1) covering an area of 17,000 hectares (Instituto Florestal 2006), and altitudes ranging from 870 to 1,100 meters (Tabarelli and Mantovani 1999). The vegetation is defined as a dense montane humid forest (‘Floresta Ombrófila Densa Montana’) (Veloso et al. 1991) and the annual precipitation is about 2200 mm. The annual mean temperature varies from 18°C to 22°C.

### Field work

Small mammals were sampled by commercial live-traps (Sherman and Tomahawk-like traps) and pitfall-traps. In July 2008, a pilot experiment was performed from one to three nights, with a total sampling effort of 300 live-traps/night. From September 2008 to September 2009, field survey was carried out bimonthly during five consecutive nights. During this period, we set up six grids with 30 live-traps per grid and 12 transects of pitfall-traps. Live-traps were arranged in a 0.6 ha grids (60 × 100 m each) with 24 trap stations spaced every 20 meters. Each trap station received one Sherman of different size, randomly set (small, 25 × 7.5 × 9.5 cm; medium, 30 × 7.5 × 9.5 cm; large, 37.5 × 10 × 12 cm; H.B. Sherman Trap®, Inc., Tallahassee, Florida, USA). We also set randomly a Tomahawk-like trap (45 × 16 × 16 cm; Rosaminas Serviço Engenharia e Comércio Ltda. Piraúba, Minas Gerais, Brazil) at six trapping stations. Overall, we had 6300 live-trap/night.

The 12 transects of pitfall-traps were pairwise 30 meters apart, from November 2008 to September 2009. Each transect received four plastic buckets (60L, 40 cm top diameter, 35 cm bottom diameter, and 56 cm depth) buried with the rim at ground level, spaced every 10 meters each. The buckets on each line were connected with a 0.5 meters tall plastic drift fence that extended an additional 10 meters at each end, totaling 50 meters of fence. In total, we used 48 buckets, resulting in 1,440 pitfall-traps/night.

Different sizes and models of traps were used to optimize the sampling, aiming to reduce the selectivity based on body size and/or habits of the animals. Attractive baits (mashed bananas, peanut butter, bacon and corn meal) were placed in both kinds of traps. All traps were checked daily, preferably on the first hours in the morning.

Trapping and handling were carried out under ICMBio licence (number 14428-2) of Instituto Chico Mendes de Conservação da Biodiversidade.

Animals were euthanized according to the protocol of the “Animal experimentation ethics” (Carpenter et al. 1996) and under permission of Instituto Butantan Ethics Committee (242/05). The skins, skulls and partial skeletons were deposited in the Museu de Zoologia da Universidade de São Paulo (MZUSP) (still without MZUSP number), Museu Nacional da Universidade Federal do Rio de Janeiro (MN) and Coleção de Mamíferos da Universidade Federal do Espírito Santo (UFES) (Table 1).

**Table 1.** A list of cytogenetically studied small mammals from Santa Virgínia, Parque Estadual da Serra do Mar, state of São Paulo, Brazil. N: number of individuals analyzed. Specimens voucher/Museum Field number: ROD and MARS - Laboratório de Ecologia e Evolução Instituto Butantan, Brazil; MN - Museu Nacional, Universidade Federal do Rio de Janeiro, Rio de Janeiro, Brazil; UFES - Coleção de Mamíferos da Universidade Federal do Espírito Santo, Brazil. 2n: diploid number, and FNa: number of autosomes arms. **Morphologies: A=acrocentric; M=metacentric; SM=submetacentric; ST=subtelocentric. Grey cells correspond to species-specific karyotypes**.

ORDER Family Tribe *Species*	N	Specimens voucher/ museum field number	Distribution	2n	FNa	Autosome pairs^a^	Sex chromosomes	Variable cytogenetic characteristics	Karyotype reference	Figure No.
**ORDER RODENTIA** **Family Cricetidae** Tribe Akodontini *Akodon montensis*	3♀6♂	ROD 3*, 6*, 11*, 28*, 29* UFES 2235-2237, 2239	From Rio de Janeiro to Rio Grande do Sul and Minas Gerais, Brazil ^1, 2^	24, 24 (+ 1B)	42	9 large to medium M/SM; 1 A; 1 small M	X: medium A Y: small A	X chromosome polymorphism (enlarged short arm), 1 SM B-chromosome	Kasahara and Yonenaga-Yassuda (1982)	3a
*Blarinomys breviceps*	1♀	UFES 2263	Endemic of Atlantic Forest, Brazil^1, 2^	29 (+2B)	50	11 medium M/SM 1 A Heteromorphic pair:1 M + 2 A	X: large A	Heteromorphic pair, 2 M B-chromosomes	Ventura et al. (2012)	See Ventura et al. (2012)
*Brucepattersonius soricinus*	1♀, 1♂	MN 78955, 78956	Southeastern Brazil, exclusively in Atlantic Forest^1, 2, 3^	52	52	24 medium to small A; 1 small SM	X: large ST Y: small A	-	Bonvicino et al. (1998)	3b–d
*Thaptomys nigrita*	2♂	ROD 2*, 4*	South Bahia to the north of Rio Grande do Sul, Brazil^1, 2^	52	52	24 medium to small A; 1 small SM	X: large A Y: small SM	-	Kasahara and Yonenaga-Yassuda (1984)	5a
Tribe Oryzomyini *Drymoreomys albimaculatus*	1♀, 1♂	UFES 2271, 2272	Endemic of Atlantic Forest, Brazil ^4^	62	62	29 medium to small A; 1 small M	X: large SM Y large SM, smaller than the X	-	Suárez-Villota et al. (2013)	See Suárez-Villota et al. (2013)
*Euryoryzomys russatus*	1♀, 7♂	ROD 5*, 12*, 30* UFES 2242- 2244, 2265-2266	Coastal region of Brazil from Bahia to Rio Grande do Sul^1, 2^	80	86	35 A decreasing in size; 4 small M	X: large SM Y: small A or small ST	Sex chromosomes polymorphisms	Andrades-Miranda et al. (2000)	5b
*Nectomys squamipes*	1♀	UFES 2270	Eastern Brazil ^2^	56 (+2B)	56	26 A decreasing in size; 1 small M	X: large SM	2 small SM B-chromosomes	Silva and Yonenaga-Yassuda (1998)	4a
*Oligoryzomys nigripes*	4♀, 4♂	ROD 34*, UFES 2274-2280	From South Bahia to Rio Grande do Sul, Brazil^1, 2^	62	80–82	11 M/SM decreasing in size; 19 A decreasing in size	X: large SM or large M Y: medium M or medium SM	Pericentric inversions in pair 3, sex chromosomes polymorphisms	Paresque et al. (2007)	4b
*Sooretamys angouya*	1♀, 4♂	UFES 2262, 2282-2285	From Espírito Santo to Santa Catarina, Brazil^2^	58	60	26 A decreasing in size; 2 small M	X: large A Y: medium A	-	Andrades-Miranda et al. (2000)	5c
Tribe Phyllotini *Calomys tener*	1♂	UFES 2264	Widespread in the state of São Paulo, Brazil^1, 2^	66	66	31 medium to small A; 1 M	X: large SM Y: medium A	-	Mattevi et al. (2005)	6a
Tribe Thomasomyini *Rhipidomys itoan*	1♀	UFES 2281	PESM^5, 6^	44	50	17 A decreasing in size; 1 medium SM; 3 small M	X: large SM	-	Pinheiro and Geise (2008); Costa et al. (2011)	4c
*Incertae sedis* *Juliomys pictipes*	3♂	UFES 2267-2269	Minas Gerais to Rio Grande do Sul, Brazil^1, 2^	36	34	17 A decreasing in size	X: medium A Y: small A	-	Bonvicino and Otazu (1999)	6b–c
**Family Echimyidae** *Trinomys iheringi*	2♀, 1♂	ROD 7*, 10*, UFES 2286	West of Rio de Janeiro, São Paulo to north of Paraná, Brazil^2, 7^	60+1B, 60+4B	116	29 M or SM decreasing in size	X: large SM Y: small SM	1 or 4 dot-like B-chromosomes; Secondary constriction on pair 7	Yonenaga-Yassuda et al. (1985)	4d
**ORDER DIDELPHIMORPHIA** **Family Didelphidae** *Marmosops incanus*	2♀, 1♂	MARS 1*, 5*, 6*	Eastern Brazil^8^	14	24	6 SM decreasing in size	X: small SM Y: small A	-	Carvalho et al. (2002)	7a
*Micoureus paraguayanus*	1♀, 1♂	MARS 3*, 4*	Atlantic Forest; Eastern Brazil, until Rio Grande do Sul state^8^	14	20	4 M or SM 2 A	X: medium A Y: medium A, smaller than X	-	Pereira et al. (2008)	7b
*Monodelphis scalops*	1♂	MN 78961	Espírito Santo, Rio de Janeiro and São Paulo, Brazil^8^	18	30	4 SM 3 ST 1 A	X: small ST Y: minute A	-	Present study	**2**
*Philander frenatus*	1♀, 1♂	UFES 2287-2288	From Bahia to Santa Catarina, Brazil^8^	22	20	10 A	X: medium A Y: small A		Pereira et al. (2008)	7c

Geographic distribution according to: 1. Musser and Carleton (2005); 2. Bonvicino et al. (2008); 3. Bonvicino et al. (1998); 4. Percequillo et al. (2011); 5. De Vivo et al. (2011); 6. Pinheiro and Geise (2008); 7. Woods and Kilpatrick (2005); 8. Gardner (2005). *Specimens voucher deposited in Museu de Zoologia da Universidade de São Paulo (MZUSP) without catalog number yet. **ª** Autosomal morphologies do not include Bs.

The nomenclature used in this work follows Gardner (2005), Musser and Carleton (2005), Weksler et al. (2006) and Percequillo et al. (2011). External morphologic traits of marsupials were compared with voucher specimens preserved at MZUSP.

### Chromosome preparation

Metaphases were obtained from bone marrow and spleen after *in vivo* injection of a 0.1% colchicine solution (1mL/100g of weight). Cells were suspended in 0.075M KCl solution for 20 minutes at 37°C and fixed in three washes of methanol: acetic acid (3:1). GTG and CBG-banding were performed according to Seabright (1971) and Sumner (1972), respectively. At least 20 metaphases per individual were analyzed to define the diploid number (2n) and fundamental number of autosome arms (FNa). Chromosomes were measured using the program ImageJ version 1.46 (Rasband 2011) to establish the fundamental number, according to Levan et al. (1964). Karyotypes were set up according to the literature, when available.

Specimen identification was carried out through a comparison of our data with previous cytogenetic information, external morphological characteristics, and geographic distribution (see Table 1 references).

## Results

A total of 706 small mammal specimens were captured (600 rodents and 106 marsupials) and 54 specimens were selected for chromosome preparations (46 rodents and 8 marsupials, Table 1).

On the whole, 13 species of rodents belonging to two families were cytogenetically analyzed (Table 1): *Akodon montensis* Thomas, 1913; *Blarinomys breviceps* (Winge, 1887); *Brucepattersonius soricinus* Hershkovitz, 1998; *Thaptomys nigrita* (Lichtenstein, 1829); *Drymoreomys albimaculatus* Percequillo, Weksler & Costa, 2011; *Euryoryzomys russatus* (Wagner, 1848); *Nectomys squamipes* (Brants, 1827); *Oligoryzomys nigripes* (Olfers, 1818); *Sooretamys angouya* (Fischer, 1814); *Calomys tener* (Winge, 1887); *Rhipidomys itoan* Costa, Geise, Pereira and Costa, 2011; *Juliomys pictipes* (Osgood, 1933) of family Cricetidae, and *Trinomys iheringi* (Thomas, 1911) of family Echimyidae.

Four marsupial species (Didelphimorphia) were karyotyped: *Marmosops incanus* (Lund, 1840); *Micoureus paraguayanus* (Tate, 1931); *Monodelphis scalops* (Thomas, 1888) and *Philander frenatus* (Olfers, 1818) (Table 1).

### First cytogenetic information for *Monodelphis scalops*

Eight individuals were collected, although only one male had been cytogenetically studied. Morphological data and geographic distribution comparisons allow us to identify all as *Monodelphis scalops*. The morphological traits of these individuals are similar to voucher specimens of *Monodelphis scalops* preserved at MZUSP under catalogue numbers 1528, 30702, 30712 and 30757. This species has also been reported in São Paulo state, Brazil (Gardner 2005), agreeing to our collecting site (Fig. 1).

**Figure 2.**
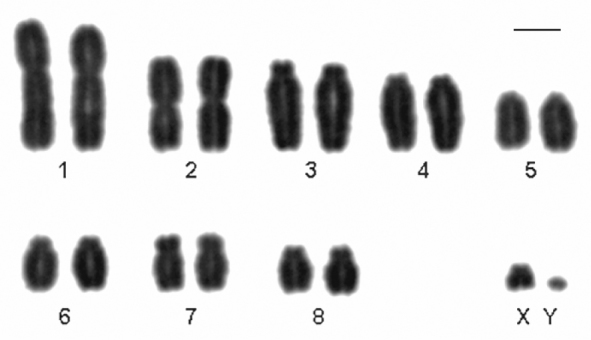
Conventional stained karyotype of *Monodelphis scalops* (2n=18, FNa=30, male). Bar = 10µm.

Here we present, for the first time, the karyotype of *Monodelphis scalops*. The karyotype of a male showed 2n=18, FNa=30. Pair 1 is a large submetacentric, pair 2 is a medium metacentric, pairs 3, 4 and 6 are medium subtelocentric, pair 5 is a medium acrocentric and pairs 7 and 8 are medium submetacentric. X chromosome is a small subtelocentric, and the Y is a minute acrocentric (Fig. 2). The short arm of pairs 4 and 6 are difficult to see depending on the condensation of the chromosome and so it was necessary to analyze and measure more than 30 metaphases to define their morphology.

### New records for PESM

**Figure 3.**
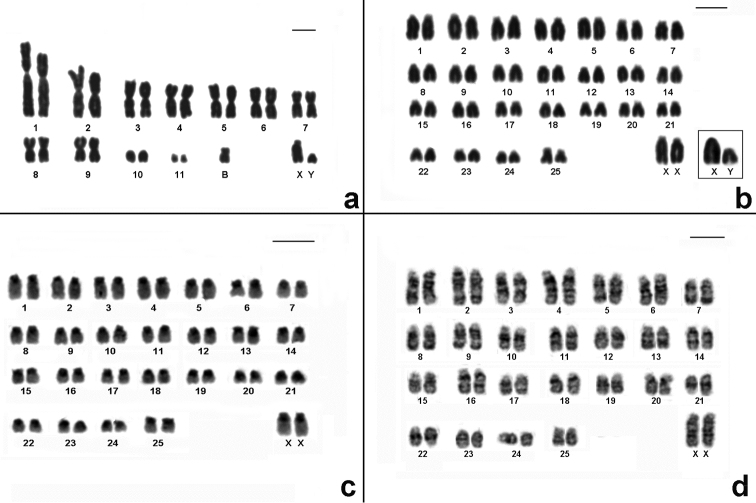
Karyotypes of the new records for PESM. **a** Conventional stained karyotype of *Akodon montensis* (2n=24+1B, FNa=42, male) **b** Conventional stained karyotype of *Brucepattersonius soricinus* (2n=52, FNa=52, female). Inset: sex chromosomes of a male **c** CBG-banding pattern of *Brucepattersonius soricinus* (2n=52, FNa=52, female) **d** GTG-banding pattern of *Brucepattersonius soricinus* (2n=52, FNa=52, female). Bar = 10µm.

Cytogenetic data helped us to report for first time the presence of *Akodon montensis*, and *Brucepattersonius soricinus* in PESM. Cytogenetic information of these species are shown in Fig. 3, Table 1. Briefly, *Akodon montensis* showed 2n=24, 25 (24+1B), FNa=42 and one individual showed a heteromorphic X chromosome with an enlarged short arm. We also detected one small supernumerary submetacentric (B) in three out of nine individuals analyzed (Fig. 3a).

*Brucepattersonius soricinus* had 2n=52, FNa=52 (Fig. 3b) and this is the first time that banding-pattern is presented in this species. The CBG-banding pattern in the female specimen showed rather pronounced amount of pericentromeric heterochromatin in all chromosomes (Fig. 3c). GTG-banding allowed the identification of all autosomic pairs and X chromosomes (Fig. 3d).

### Chromosomal variability and species-specific karyotypes

**Figure 4.**
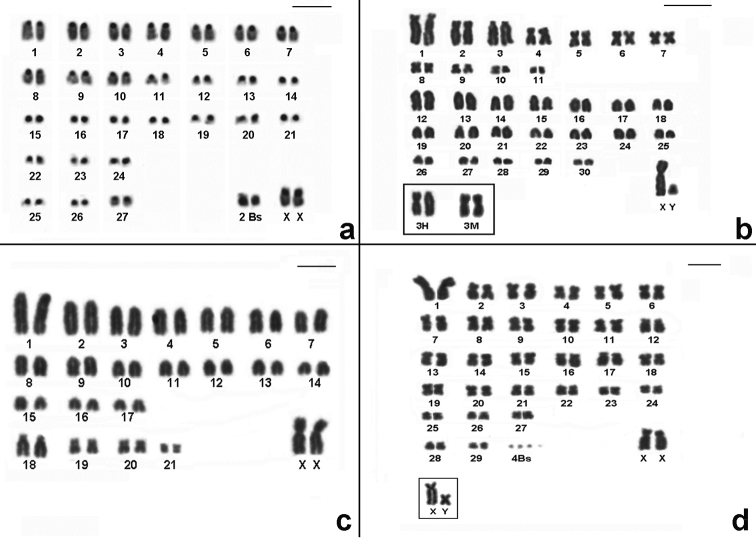
CBG-banding pattern of *Nectomys squamipes* (2n=56 + 2B, FNa=56, female) **b** Conventional stained karyotype of *Oligoryzomys nigripes* (2n=62, FNa=80, male). Inset: different forms of pair 3: heteromorphic (3H) and homomorphic metacentric (3M) **c** Conventional stained karyotype of *Rhipidomys itoan* (2n=44, FNa=50, female) **d** Conventional stained karyotype of *Trinomys iheringi* (2n=60+4Bs, FNa=116, female). Inset: sex chromosomes of a male. Bar = 10µm.

**Figure 5.**
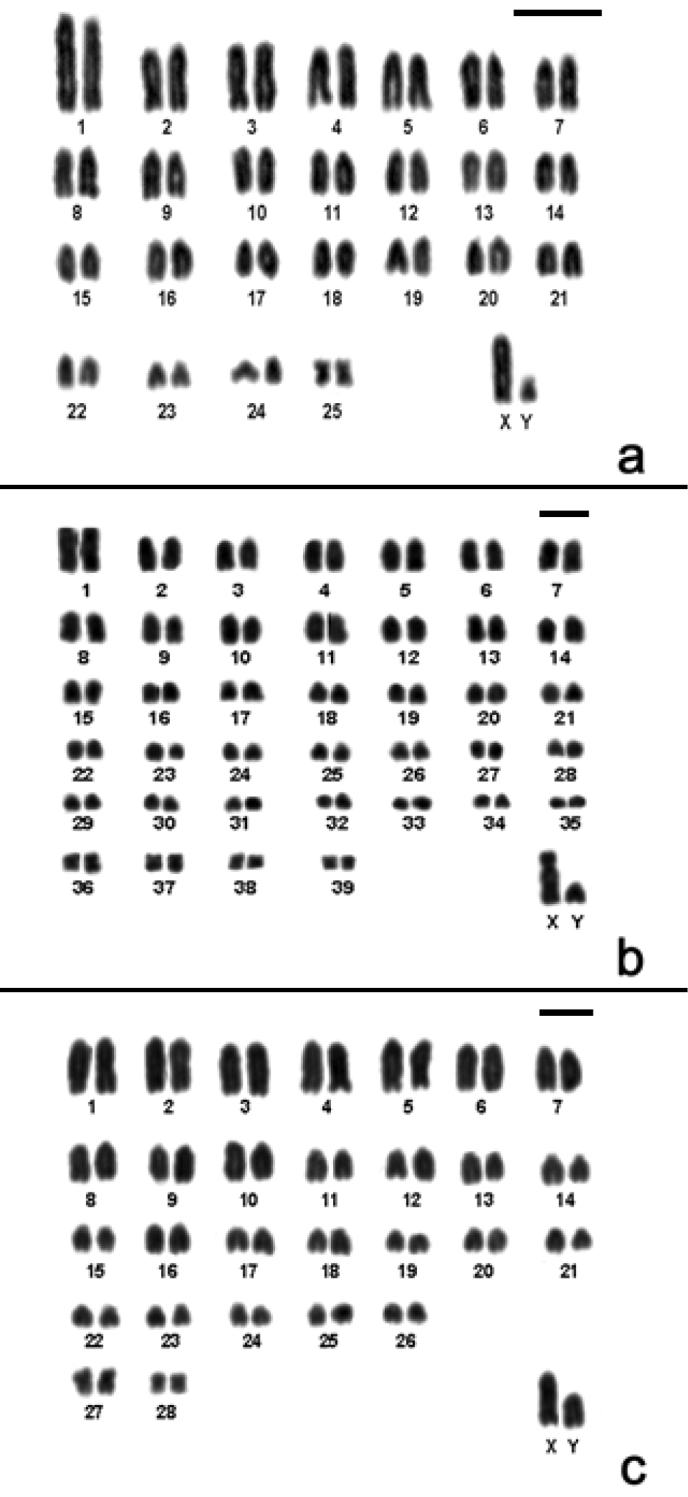
Conventional stained karyotypes: **a** *Thaptomys nigrita* (2n=52, FNa=52, male) **b** *Euryoryzomys russatus* (2n=80, FNa=86, male) **c** *Sooretamys angouya* (2n=58, FNa=60, male). Bar = 10µm.

**Figure 6.**
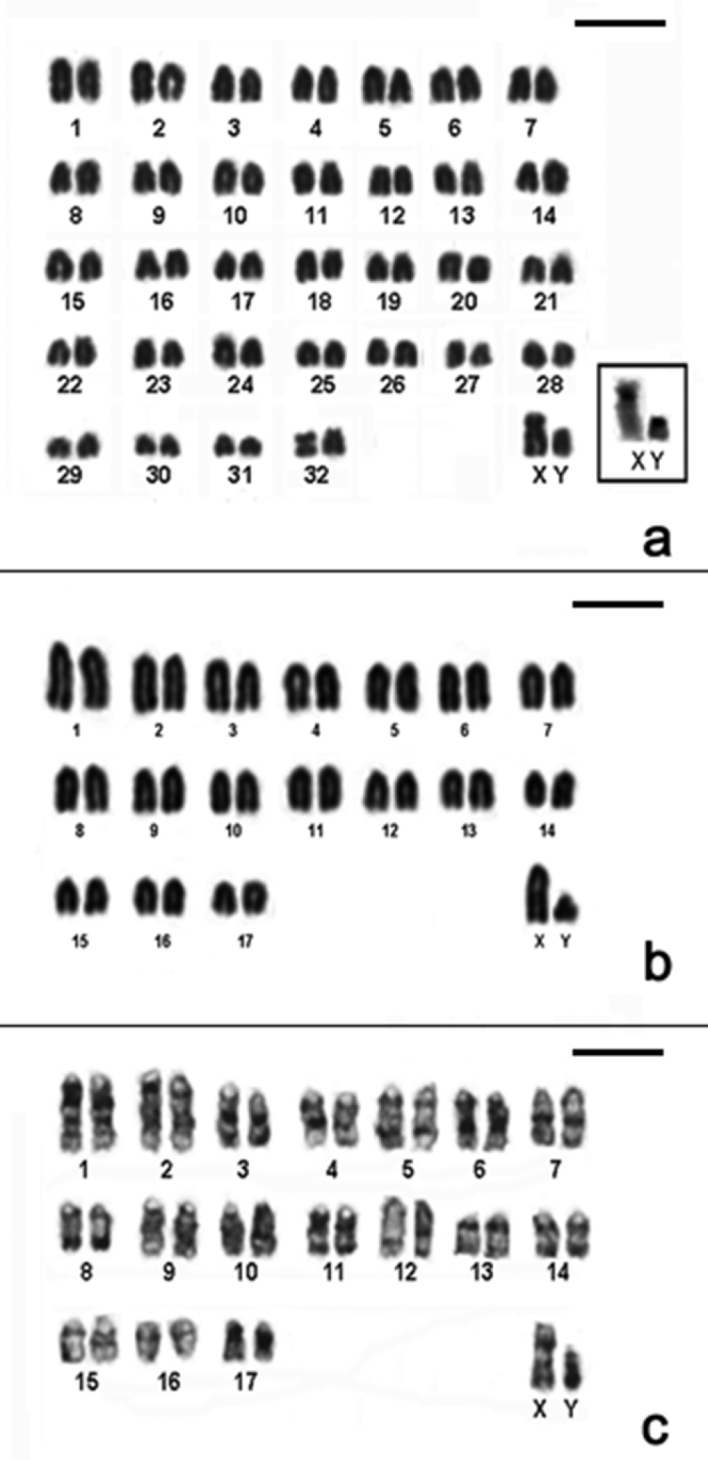
**a** Conventional stained karyotype of *Calomys tener* (2n=66, FNa=66, male). Inset: Sex chromosomes CBG-banded **b** Conventional stained karyotype of *Juliomys pictipes* (2n=36, FNa=36, male) **c** GTG-banding pattern of *Juliomys pictipes* (2n=36, FNa=36, male). Bar = 10µm.

**Figure 7.**
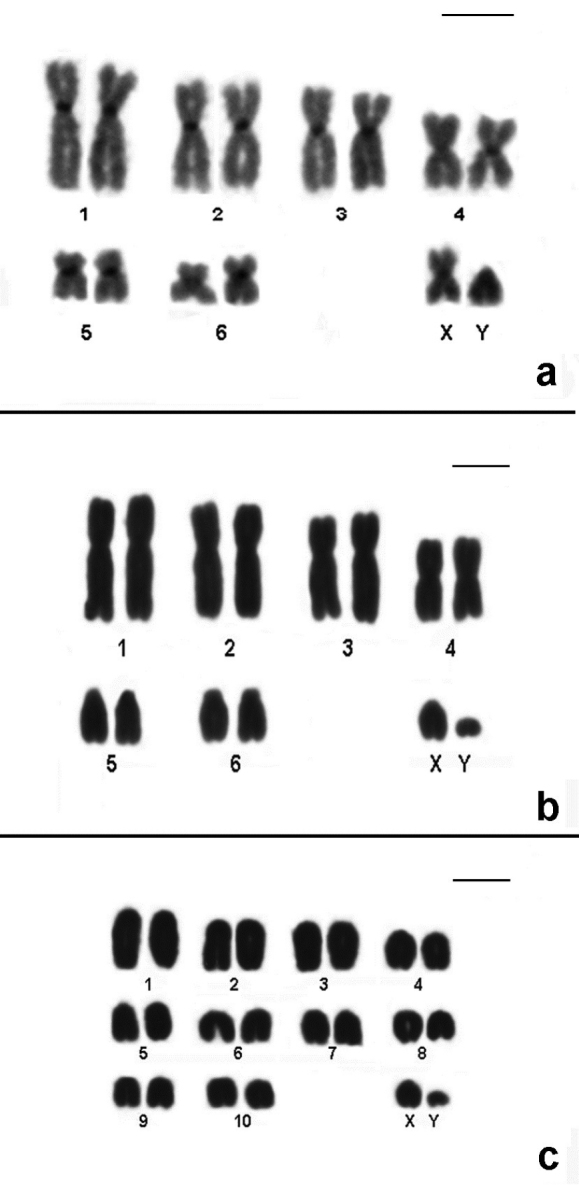
**a** CBG-banding pattern of *Marmosops incanus* (2n=14, FNa=24, male) **b** Conventional stained karyotype of *Micoureus paraguayanus* (2n=14, FNa=20, male) **c** Conventional stained karyotype of *Philander frenatus* (2n=22, FNa=20, male). Bar = 10µm.

The remaining species studied in this work have already been recorded in PESM and their karyotypes are in accordance to the literature. Karyotype information of all species analyzed and the chromosomal variability found in this work is shown in Table 1 and Figs 4–7.

Seven out of the 13 rodent species showed species-specific karyotypes: *Akodon montensis*, *Drymoreomys albimaculatus*, *Oligoryzomys nigripes*, *Sooretamys angouya*, *Calomys tener*, *Juliomys pictipes* and *Trinomys iheringi* (grey cells in Table 1). The identification of the remaining species (*Blarinomys breviceps*, *Brucepattersonius soricinus*, *Thaptomys nigrita*, *Euryoryzomys russatus*, *Nectomys squamipes*, and *Rhipidomys itoan*) required additional morphological and molecular investigation and geographic distribution information (Table 1).

Marsupials presented conserved diploid numbers of 14, 18 and 22 and were identified here by external morphological comparisons.

## Discussion

### Importance of cytogenetic study for Neotropical rodents

We proved the cytogenetic analyses as a taxonomic tool, since 7 out of 13 rodent species present species-specific karyotypes (53.8%). Besides, we identified 94% of all species, when cytogenetic data were combined with information of external morphology and geographical distribution (Table 1).

Cryptic species are relatively common in some Neotropical rodent groups and cytogenetic information was indispensable for identifying such species. For instance, *Akodon montensis* is morphologically indistinguishable from *Akodon cursor* (Winge, 1887) and both species occur in sympatry in the Atlantic Forest (Christoff et al. 2000). In addition, the occurrence of *Akodon cursor* previously recorded in Santa Virgínia/PESM (Instituto Florestal 2006) was doubtful till this study, as we proved the occurrence of *Akodon montensis* by karyotypic analysis.

Another cryptic species case occurs in the genus *Thaptomys*. *Thaptomys* sp. (2n=50) and *Thaptomys nigrita* (2n=52) are morphologically identical, so the karyotypes are the diagnostic information to distinguish both species (Ventura et al. 2004, 2010).

By contrast, *Thaptomys nigrita* and *Brucepattersonius soricinus* present very similar karyotypes (2n=52, FNa=52) however their identification can be safely done at the level of genera by external morphological characters. An accurate observation on the karyotypes of *Brucepattersonius soricinus* and *Thaptomys nigrita* showed that the pair 1 of *Thaptomys nigrita* is the largest of the chromosome set (Fig. 5a) meanwhile *Brucepattersonius soricinus* has the pair 1 similar in size to the others of the set (Figs 3b–d). We also noticed differences regarding sex chromosome morphologies of both species (Table 1). This feature could be a diagnostic tool to differentiate each karyotype, but additional cytogenetic studies (including comparative and molecular cytogenetic data) are needed to support these first observations.

*Blarinomys breviceps* presents a peculiar karyotype and it could not be considered species-specific due to the great variability in 2n and FNa (Geise et al. 2008, Ventura et al. 2012). Moreover, Ventura et al. (2012) suggested the existence of more species for the monotypic genus *Blarinomys* in Atlantic Forest since molecular phylogenetic analyses showed two geographically distinct lineages.

*Euryoryzomys russatus* does not have species-specific karyotype also. *Euryoryzomys emmonsae* Musser, Carleton, Brothers and Gardner, 1998, and *Euryoryzomys nitidus* (Thomas, 1884) share the same 2n=80, NFa=86 (Bonvicino and Geise 2006). However, when cytogenetic information is combined with morphologic and geographic distribution data, *Euryoryzomys russatus* can be confirmed.

Concerning *Nectomys squamipes*, it is not possible to affirm that this species possess species-specific karyotype with classical cytogenetic data because, when compared to *Holochilus brasiliensis* (Desmarest 1819), both karyotypes are identical (Yonenaga-Yassuda et al. 1987). Nevertheless, the association of cytogenetic, geographic distribution and external morphological characters allows the recognition of *Nectomys squamipes* as occurring at PESM (Bonvicino et al. 2008). *Nectomys squamipes* was considered for years as a carrier of two basic distinct karyotypes: 2n=56 (1 to 3Bs) and 2n=52 (1 to 3Bs), and only after crossings in laboratory, Bonvicino et al. (1996) noticed that two different species could be diagnosed - *Nectomys squamipes* (2n=56) and *Nectomys rattus* (Pelzeln, 1883), (2n=52).

The karyotype of *Rhipidomys itoan* presented here (2n=44, FNa=50 Fig. 4c) is the same one as described by Zanchin et al. (1992) and Silva and Yonenaga-Yassuda (1999). Pinheiro and Geise (2008) also found an identical karyotype for a species referred as *Rhipidomys* sp., trapped in Picinguaba (PESM), and De Vivo et al. (2011) reported an undescribed species of *Rhipidomys* that occurs at the Parque Estadual da Serra do Mar. Recently, two new species from Atlantic Forest were described: *Rhipidomys tribei* Costa, Geise, Pereira and Costa, 2011 and *Rhipidomys itoan*; and the latter presented 2n=44, FNa=48, 49, 50 (Costa et al. 2011). Santa Virgínia is embedded in the geographical distribution described for this species and molecular analyzes confirmed that this sample belongs to *Rhipidomys itoan* species. Nevertheless, we do not consider this karyotype species-specific.

Finally, cytogenetic analysis was useful in identifying *Thaptomys iheringi* as two species – *Thaptomys iheringi* and *Thaptomys dimidiatus* (Günther, 1876) - occur in Atlantic Forest. Despite the regular chromosome set of *Thaptomys iheringi* (not considering B chromosomes) is identical to the one described for the species *Thaptomys dimidiatus* (2n=60, FNa=116) by Pessoa et al. (2005), the presence of at least one B and the morphology of Y chromosome in *Thaptomys iheringi* represent good characters to diagnose the species.

### Chromosome variations

Mammals have remarkable diversity in species karyotypes, and rodents exhibit noteworthy variability of diploid chromosome number (O’Brien et al. 2006, Romanenko et al. 2012). For instance, in this work, diploid numbers of rodents ranged from 24 in *Akodon montensis* to 80 in *Euryoryzomys russatus*.

The chromosome variation observed here is due to the presence of supernumerary chromosomes (B chromosomes), sex chromosome heteromorphism and/or polymorphism, as well as autosomal polymorphisms. This chromosome variability does not cause a problem in characterizing the species, except in the case of *Thaptomys iheringi*, in which the presence of at least one B chromosome is sufficient to confirm its identity.

Structural rearrangements may explain much of the observed karyotype diversity in rodents. In this regard, Robertsonian fusions/fissions (whole-arm translocations) and pericentric inversions, have long been considered the predominant rearrangements in natural populations of rodents (Patton and Sherwood 1983). Nevertheless, studies with more refined techniques such as fluorescent *in situ* hybridization and chromosome painting demonstrate that tandem fusions, reciprocal translocations, and paracentric inversions are much more common than previously thought (Hass et al. 2008, Ventura et al. 2009, Romanenko et al. 2012).

Our data showed two species with pericentric inversion rearrangements, *Oligoryzomys nigripes* and *Rhipidomys itoan*. *Oligoryzomys nigripes* showed variation in autosomal pair 3 (Fig. 4b) but this rearrangement had also been reported in pairs 2, 4 and 8, which places this species as one of the most polymorphic within Neotropical rodents (Paresque et al. 2007). The genus *Rhipidomys* frequently shows 2n=44, except for the 2n=50 reported by Silva and Yonenaga-Yassuda (1999) from Amazonas, in contrast with differences in the FNa (Zanchin et al. 1992, Costa et al. 2011). The variation of FNa, which represents the commonest chromosome change observed for the genus, may be a consequence of pericentric inversion events.

Karyotype diversity is also enhanced in mammals due to the presence of B chromosomes. B chromosomes are extra elements found in the karyotypes of many eukaryotic species. Their functions and molecular composition remain obscure but, apparently in mammals, these chromosomes neither promote phenotypic alterations nor affect fitness of individuals (Jones and Rees 1982, Trifonov et al. 2010). B chromosomes are known in nine Brazilian rodent species (Silva and Yonenaga-Yassuda 2004, Ventura et al. 2012). Herein, we found B chromosomes in four out of 13 species of rodents (30,76%, i.e. almost a third of the total): *Akodon montensis*, *Brucepattersonius breviceps*, *Nectomys squamipes* and *Thaptomys iheringi*. Silva and Yonenaga-Yassuda (2004) found B chromosomes in *Sooretamys angouya* (referred at that time as *Oryzomys angouya*), however, in our sample, B chromosomes were not observed for this species (Fig. 5c).

Sex chromosome heteromorphisms/polymorphisms were found in *Akodon montensis* and *Oligoryzomys nigripes*, and the variation is due to addition/deletion of constitutive heterochromatin, as described by Kasahara and Yonenaga-Yassuda (1982) and Paresque et al. (2007), respectively.

### Marsupials

Cytogenetic data exposed three diploid numbers for the family Didelphidae: 2n=14, 18 and 22 (Reig et al. 1977, Carvalho et al. 2002). As the karyotypes of American marsupials are conserved, cytogenetic analyses cannot be considered as a diagnostic tool to identify species. However, differences in banding patterns could help in the characterization of some taxa, for instance, *Marmosops incanus* (Svartman 2009).

In the present paper we report for the first time the karyotype of *Monodelphis scalops* which is similar to the one described for *Monodelphis kunsi* Pine, 1975 and *Monodelphis brevicauda* (Erxleben, 1777) by Carvalho et al. (2002), except for the morphology of the sex chromosomes (Fig. 2). Besides, *Monodelphis scalops* karyotype differs from *Monodelphis rubida* (Thomas, 1899) (2n=18, FNa=32) (Pereira et al. 2008) due to the presence of one acrocentric pair (#5) instead of a biarmed pair (Fig. 2).

## Final considerations

Our species list is an evidence of the limited knowledge of small mammals in PESM since the karyotype of *Monodelphis scalops* is reported for the first time and *Akodon montensis* and *Brucepattersonius soricinus* are new records for the park. According to De Vivo et al. (2011), it is important to increase samples in areas of dense humid forest since these areas are poorly surveyed. The number of species collected during the period of 14 months in Santa Virgínia should be considered highly representative, and this effort brought to light new findings. This includes the specimen of *Blarinomys breviceps* herein mentioned which was added to a larger sample with animals collected from different localities of Brazil and the diploid numbers ranged from 28 to 52 (Ventura et al. 2012), as well as *Drymoreomys albimaculatus* which was studied by Suárez-Villota et al. (2013).

The improvements to the list of mammals of PESM could be attributed to different methods of capture (live and pitfall traps) to enhance the success of trapping in different habitats. The multidisciplinary approach employed is also evidently important in some cases as presented above. Additionally, data on diversity and geographical distribution of species are essential to reach conservation strategies, and the significance of Santa Virgínia / PESM in the preservation of the Neotropical fauna becomes more clear.
