# Supplementary figures and images for: New karyologycal data and cytotaxonomic considerations on small mammals from Santa Virgínia (Parque Estadual da Serra do Mar, Atlantic Forest, Brazil)
Source: Comp Cytogenet. 2014 Jan 24;8(1):11–30. doi: 10.3897/CompCytogen.v8i1.6430 (PMC3978240; doi:10.3897/CompCytogen.v8i1.6430)

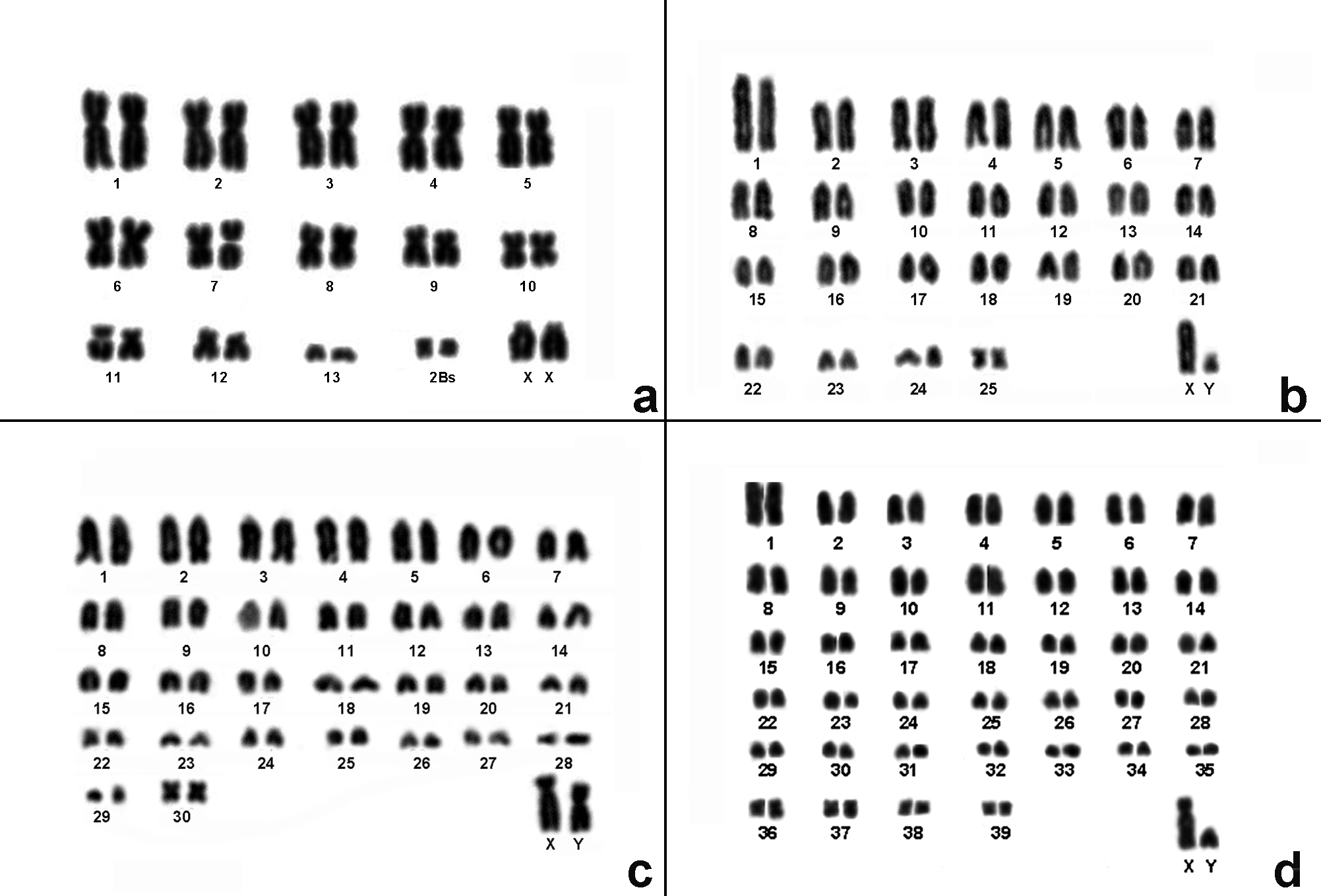

Supplement: Figure S1 [file CompCytogen-008-011-s001.tif]

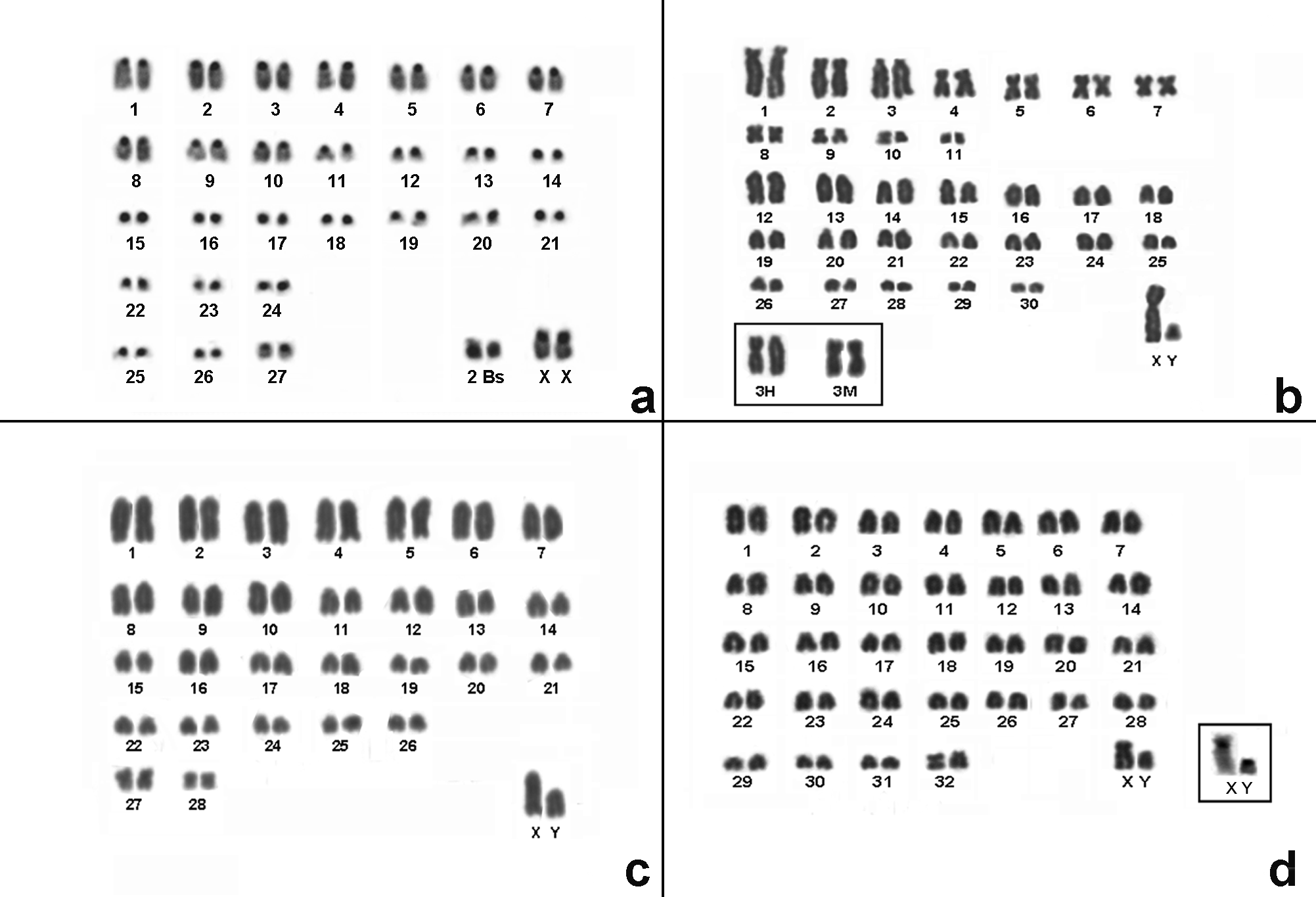

Supplement: Figure S2 [file CompCytogen-008-011-s002.tif]

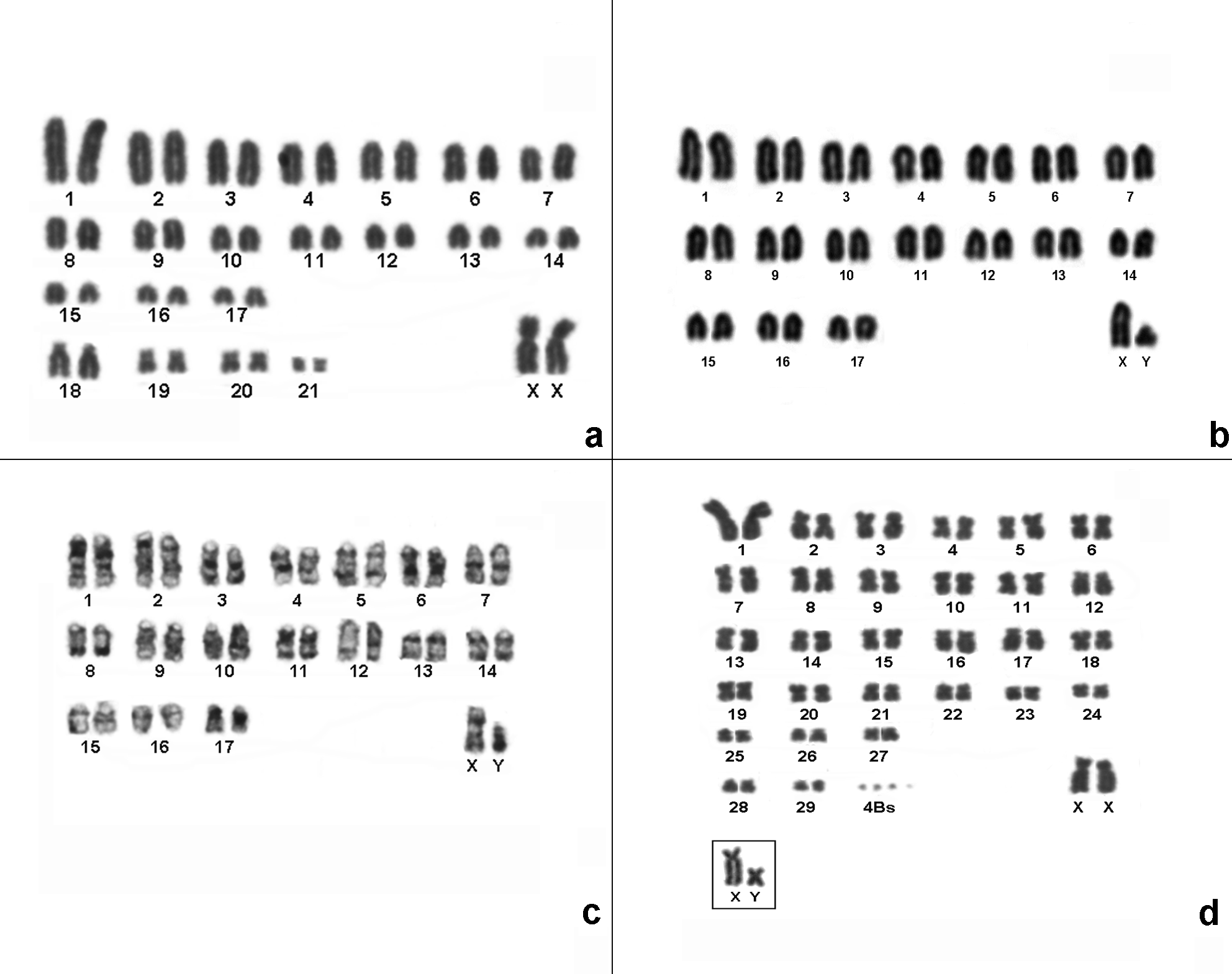

Supplement: Figure S3 [file CompCytogen-008-011-s003.tif]

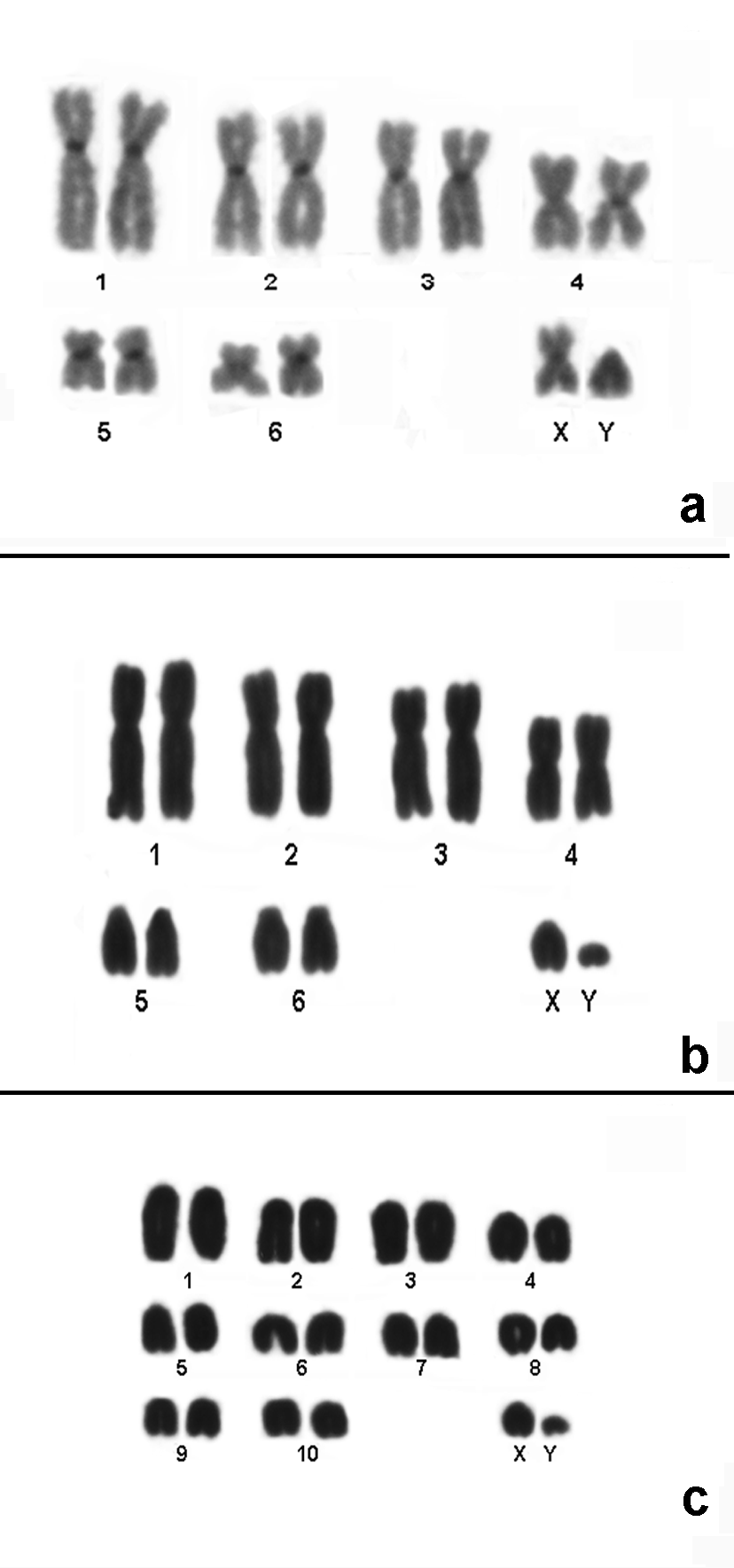

Supplement: Figure S4 [file CompCytogen-008-011-s004.tif]
